# Machine Learning Models for Identification and Prediction of Toxic Organic Compounds Using *Daphnia magna* Transcriptomic Profiles

**DOI:** 10.3390/life12091443

**Published:** 2022-09-16

**Authors:** Tae-June Choi, Hyung-Eun An, Chang-Bae Kim

**Affiliations:** Department of Biotechnology, Sangmyung University, Seoul 03016, Korea

**Keywords:** environmental monitoring, aquatic ecosystem, toxic organic compounds, *Daphnia magna*, transcriptomic profiles, machine learning, random forest

## Abstract

A wide range of environmental factors heavily impact aquatic ecosystems, in turn, affecting human health. Toxic organic compounds resulting from anthropogenic activity are a source of pollution in aquatic ecosystems. To evaluate these contaminants, current approaches mainly rely on acute and chronic toxicity tests, but cannot provide explicit insights into the causes of toxicity. As an alternative, genome-wide gene expression systems allow the identification of contaminants causing toxicity by monitoring the organisms’ response to toxic substances. In this study, we selected 22 toxic organic compounds, classified as pesticides, herbicides, or industrial chemicals, that induce environmental problems in aquatic ecosystems and affect human-health. To identify toxic organic compounds using gene expression data from *Daphnia magna*, we evaluated the performance of three machine learning based feature-ranking algorithms (Learning Vector Quantization, Random Forest, and Support Vector Machines with a Linear kernel), and nine classifiers (Linear Discriminant Analysis, Classification And Regression Trees, K-nearest neighbors, Support Vector Machines with a Linear kernel, Random Forest, Boosted C5.0, Gradient Boosting Machine, eXtreme Gradient Boosting with tree, and eXtreme Gradient Boosting with DART booster). Our analysis revealed that a combination of feature selection based on feature-ranking and a random forest classification algorithm had the best model performance, with an accuracy of 95.7%. This is a preliminary study to establish a model for the monitoring of aquatic toxic substances by machine learning. This model could be an effective tool to manage contaminants and toxic organic compounds in aquatic systems.

## 1. Introduction

Freshwater habitats worldwide are heavily influenced by a wide range of abiotic environmental factors [1,2]. Pollution of aquatic ecosystems by anthropogenic compounds is a major environmental issue; therefore, it is important to discover means for early detection and environmental protection. Among them, toxic organic compounds, such as pesticides, herbicides, and industrial chemicals, can cause environmental problems in aquatic ecosystems and can affect human health through contamination of drinking water. For example, pesticides can reduce the diversity and abundance of plants and insects in habitats and the food available to fish and other aquatic organisms [3]. In addition, the presence of industrial intermediates, such as chloroform and phenol, in drinking water is a potential human health concern [4].

Evaluating the effects of environmental factors on aquatic ecosystems is primarily conducted through acute and chronic toxicity testing. Although these approaches can detect the presence of toxins in the field, it is difficult to identify the underlying factors. In addition, the detection and assessment approaches rely on various processing and classification procedures, with repeated toxicity tests to identify key toxic substances [5]. These approaches can provide important information; however, they are time-consuming, labor-intensive, and expensive, and are often unable to identify the cause of toxicity [1]. With the development of next-generation sequencing (NGS), gene expression profiles enable the rapid assessment of contaminants in environmental samples and provide a robust and cost-effective alternative to traditional methods [6]. Changes in gene expression are an early response to environmental stress. Gene expression is sensitive to environmental cues and has been shown to exhibit specific patterns under various conditions [7]. Additionally, batch gene expression data contain the expression levels of thousands of genes evaluated in various conditions. As a result, gene expression analysis allows the identification of toxic substances in aquatic ecosystems, and the monitoring of them through early diagnosis [8,9].

High-throughput gene expression analysis, such as microarray technology and RNA sequencing, generates tremendous amounts of data. Conventionally, statistical methods are used for comparative analysis of gene expression data; however, the identification and classification of feature genes requires sophisticated computational approaches [10]. Machine learning (ML) is an artificial intelligence-based approach that can automatically learn from data and construct a system with enhanced performance. ML techniques have been broadly used to analyze microarray data as they can analyze high-dimensional gene expression data [11,12]. Ref. [1] applied a RF classification to *Daphnia magna* gene expression data for 36 chemical exposures and developed predictive models of contaminant exposure [1]. Similarly, ML approaches have been applied to predict ecological risks. For example, Ref. [13] predicted toxic endocrine-disrupting chemicals in aquatic species using a support vector machine algorithm with a nonlinear algorithm [13].

Model organisms, such as *Daphnia* species that exhibit a wide geographic distribution and are highly sensitive to environmental factors, have been used to assess the effects of abiotic factors on freshwater ecosystems [14,15]. *Daphnia magna* is widely used in aquatic toxicology to estimate the effects of chemicals on aquatic ecosystems. It has a short generation time and life cycle and is easy to culture and test under controlled laboratory conditions. Furthermore, it is a standard test organism for toxicity evaluation, as designated by the Organization for Economic Co-operation and Development (OECD). In addition, many studies have performed gene expression profiling (by microarray) in *D. magna* following exposure to toxic organic compounds, based on OECD guidelines [16,17]. Therefore, we obtained the transcriptomic profiles (by microarray) for *D. magna*.

In this study, an ML approach, based on a combination of feature-selection and classifier algorithms, was used to construct an optimized model to identify and predict the toxic organic compounds (e.g., pesticides, herbicides, and industrial chemicals) using open-source microarray data from *D. magna*. Here, we built a model for identification and prediction of toxic organic compounds by evaluating and comparing nine different algorithms: one simple linear, three nonlinear, and five ensemble algorithms. Our findings can serve as a reference to identify and predict various environmental factors for aquatic environment monitoring.

## 2. Materials and Methods

### 2.1. Dataset Collection

The National Center for Biotechnology Information Gene Expression Omnibus (GEO) (https://www.ncbi.nlm.nih.gov/geo, accessed on 26 October 2021) database was asked to search for gene expression data (by microarray) studies for *D. magna* [18]. Studies focusing on exposure to toxic organic compounds causing environmental problems were selected. The datasets used in this study are shown in Table 1. The datasets (GEO accession number) GSE43564 [1], GSE55132 [19], GSE43960 [2], and GSE45053 [20] were retrieved and consisted of 16, 1, 1, and 4 toxic organic compounds, respectively. In each study, all replicate data on exposure to organic compounds were used for learning. Although each dataset had different exposure conditions to organic compounds, the gene expression data used to build the model were obtained after exposure to sublethal concentrations of organic compounds (Supplementary Table S1). The dataset used for ML were constructed as the normalized gene expression level obtained after exposure (experiment) to organic compounds against that of a gene not exposed (control); low-expression genes were removed. In total, 13,481 genes (features) were used for each dataset (Supplementary Table S2).

### 2.2. Feature-Selection

The Classification and Regression Training package in the statistical environment R (v4.1.1) was used for feature selection, classifier algorithm evaluation, and to build a model able to identify toxic organic compounds [21,22,23]. Similar to other gene expression profiling studies with hundreds of genes (features), many genes used as predictors were highly correlated with one another. However, numerous gene expression data require a feature selection process to select only important features (genes) which can identify toxic organic compounds to avoid collinearity, reduce data dimensionality, and minimize noise [24]. To this end, we considered two feature selection methods: (1) removal of redundant features and (2) ranking features by importance (feature-ranking) using three algorithms (Table 2).

### 2.3. Training of Classification Algorithms

Importance-ranked features were used to evaluate nine classification algorithms (classifiers) for identification of toxic organic compounds. Combinations of the feature-selection methods and nine classification algorithms were examined by implementing a 10-fold cross-validation procedure with three repeats, which is a standard validation technique. The 10-fold cross-validation steps were used to randomly divide the data used for modeling into 10 parts, nine of which were used as training data, and the remaining data were used for validation. Each validation result had a corresponding accuracy value, and the average of 10 validations was used to evaluate model accuracy. Feature selection methods were also used to perform a 10-fold cross-validation procedure with three repeats to improve the performance of the final model. The classifiers included nine algorithms from three different families: linear, nonlinear, and ensemble (Table 3). Linear classifiers included Linear discriminant analysis (LDA). Nonlinear classifiers included classification and regression trees (CART), K-nearest neighbors (Knn), and support vector machines with a linear kernel (SVML). The ensemble models comprised RF, Boosted C5.0, gradient boosting machine (GBM), and eXtreme gradient boosting (XGBoost): xgbTree and xgbDART. While most algorithms used default parameters, the tuning parameters in XGBoost models were set to control overfitting: nrounds = 50, max_depth = 3, eta = 0.3, gamma = 0, min_child_weight = 1, colsample_bytree = 1, subsample = 1, and the parameters rate_drop = 0, skip_drop = 0 were added to xgbDART.

To estimate the accuracy of the classification algorithms, the dataset was randomly divided into training and test sets at a 7:3 ratio. The classification algorithms were then evaluated on the training set to build a model for identification of toxic organic compounds. A boxplot of the model evaluation results compared the mean accuracy and dispersion of each model. After selecting the model with the highest accuracy, the test set was used for validation. Finally, the prediction results were summarized in a confusion matrix, and the accuracy (1), sensitivity (2), and specificity (3) for all toxic organic compounds were analyzed using the following formulae, where TP is the true positive, TN is the true negative, FP is the false positive, and FN is the false negative:

(1) $$\mathrm{Accuracy}=\frac{\mathrm{TP}+\mathrm{TN}}{\mathrm{TP}+\mathrm{FP}+\mathrm{TN}+\mathrm{FN}}$$

(2) $$\mathrm{Sensitivity}=\frac{\mathrm{TP}}{\mathrm{TP}+\mathrm{FN}}$$

(3) $$\mathrm{Specificity}=\frac{\mathrm{TN}}{\mathrm{TN}+\mathrm{FP}}$$

## 3. Results

The redundant feature-selection method removed 4058 of 13,481 features, leaving 9423 features (Supplementary Table S3). The 20 most important features were ranked using three algorithms with 10-fold cross-validation and used to evaluate nine classification algorithms. The features selected using the three algorithms showed different features (Supplementary Table S4). Therefore, the features selected by each algorithm were used to evaluate the classifier, and compared for model optimization.

### 3.1. Comparison of Feature-Selection Method and Classification Algorithm Combinations

**Figure 1:**
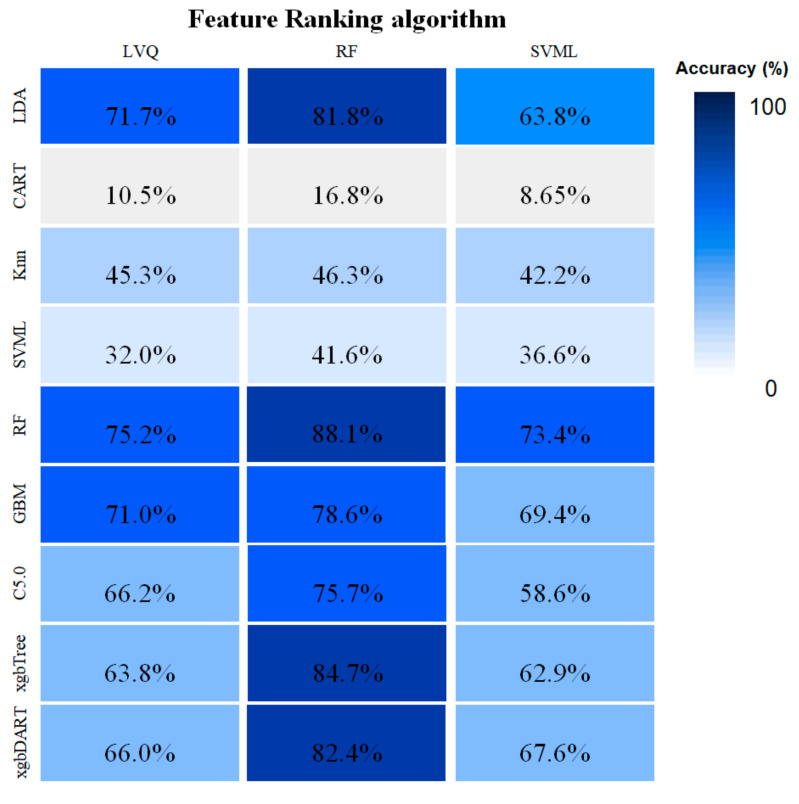
Model accuracy of each feature-ranking/classifier algorithm combination with three repetitions of a 10-fold cross-validation step. Colors from dark blue to white indicate high to low accuracy, respectively.

To build an optimized model that best identified toxic organic compounds, we combined and evaluated the nine classifiers against the three different feature-ranking algorithms and compared each combination (Figure 1). In combination with the three feature-ranking algorithms, linear and ensemble classifiers showed relatively higher accuracy than nonlinear classifiers. Among the three feature-ranking algorithms, the RF feature-ranking algorithm showed the highest accuracy against all classifiers (from 73.4% in SVML to 88.1% in RF classifier); the model combining a RF classifier and a feature-ranking algorithm showed 88.1% accuracy. In contrast, the CART classifier had the lowest accuracy among feature-ranking algorithms (from 8.5% in SVML to 16.8% in RF feature-ranking algorithms).

### 3.2. Model Evaluation Using the Test Set

**Figure 2:**
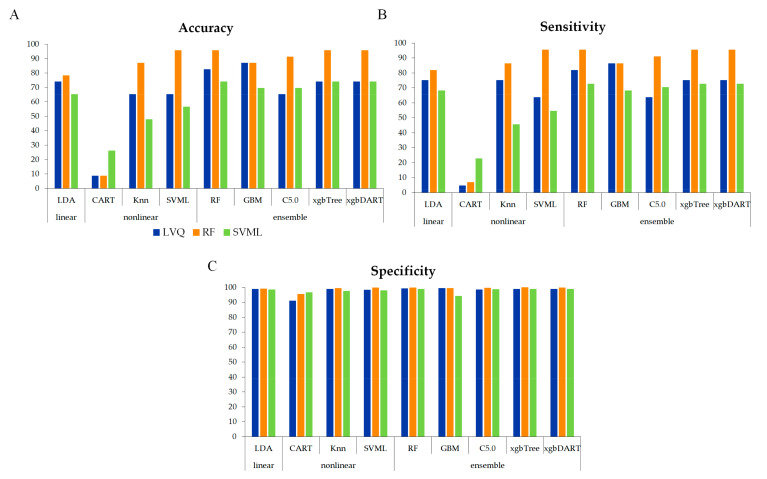
Performance of predictive models according to feature-ranking and classifier algorithm combinations: (**A**) accuracy. (**B**) sensitivity. (**C**) specificity.

To build an optimal model, the performances of each model, based on combinations of feature-ranking and classifier, were evaluated using an independent test dataset (Figure 2). In combination with the LVQ feature-ranking algorithm, the GBM classifier showed the best performance in terms of accuracy (87.0%), sensitivity (86%), and specificity (99%). On the other hand, the CART classifier exhibited the lowest performance. The highest classification accuracy was observed with the combination of RF feature-ranking algorithms and the SVML, RF, xgbTree, and xgbDART classifiers (95.7%). With the RF feature-ranking algorithm, the SVML, RF, xgbTree, and xgbDART classifiers showed the best performance regarding accuracy (95.7%), sensitivity (95%), and specificity (99%). With an RF classifier, the SVML feature-ranking algorithm exhibited the best performance in terms of accuracy (73.9%), sensitivity (73%), and specificity (99%). The specificity values were >0.9 in all combinations of feature-ranking and classifier algorithms. Among them, the LVQ and RF feature-ranking algorithms showed the highest specificity in combination with the GBM (99%) and xgbTree classifiers (100%), respectively. The SVML feature-ranking algorithm showed the highest specificity combined with the LDA, RF, xgbTree, and xgbDART classifiers (98%). Consequently, an optimized model combining RF feature-ranking algorithm and an RF classifier accomplished 95.7% accuracy, 96% sensitivity, and 99% specificity.

**Figure 3:**
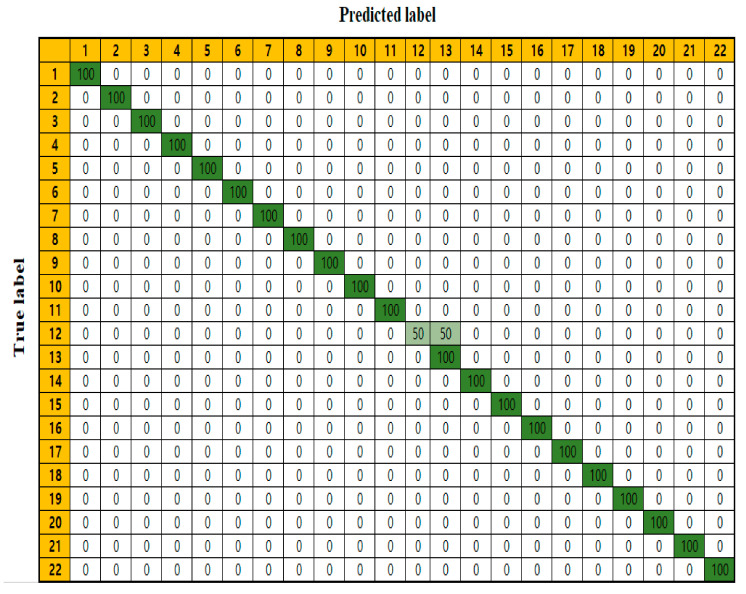
Confusion matrix for feature-ranking/classifier combinations using random forest (RF) algorithms. Colors from green to white indicate high to low accuracy, respectively.

The confusion matrix of the feature-ranking and classifier combination, based on the RF algorithms, of the predictive model with the best performance, is shown in Figure 3. The confusion matrix indicated 22 toxic organic compounds (Supplementary Table S5). Rows indicate actual toxic organic compounds, whereas columns indicate the predicted toxic organic compounds. All compounds were predicted with 100% accuracy, except fluvoxamine, misclassified as fluoxetine (50%).

## 4. Discussion

Identification of toxic pollutants in aquatic ecosystems is time-consuming, labor-intensive, and challenging. Currently, approaches such as physical isolation or monitoring of residual toxicity are used [1]. Although these approaches are primarily used for environmental monitoring, they are costly and require sorting and treatment of contaminated water for human consumption, which is time-consuming [5]. *D. magna* presents unique gene expression patterns for survival and reproduction depending on environmental factors, and these patterns can serve to examine the causes of toxicity [25,26,27]. Therefore, we applied an approach to identify the contaminants underlying toxicity based on ML, using gene expression data obtained from organisms exposed to toxic substances. This method could quickly identify the cause of toxicity in the aquatic ecosystem, even at low toxic concentrations. However, this approach requires field validation and conventional toxicity identification testing for detecting the specific toxic substances in the water. This time-saving, cost-effective combination allows precise identification of the cause of toxicity. Portable sequencers, such as nanopore sequencers that can sequence environmental DNA and RNA in the field, facilitate identification of toxic substances and gene expression profiling in aquatic ecosystems [28].

The features extracted with each selection method were differently ranked. These feature-ranking methods showed different performances according to the classification algorithm. Thus, integrating feature-selection based models into a single optimized model can overcome their restrictions and result in a more balanced model with better performance. We developed an optimized model consisting of a subset of 20 genes with gene expression profiles capable of discriminating toxic organic compounds. The feature-ranking and RF classifier showed the best performance, with 95.7% accuracy. Generally, ensemble classification algorithms, generated by integrating predictions of multiple component classifiers, exhibited good performance [29]. Previous studies reported that ensemble classification, including boosting, bagging, and stacking algorithms, often perform better than single decision trees [30,31]. In addition, there are many studies that have applied machine learning methods, such as quantitative structure–activity relationship (QSAR) models, to predict toxic substances [32,33]. However, this model is still quite limited for field use. One of the reasons is that many QSAR models do not have the “right” combination of features required for successful use. For example, several algorithms used in this study (such as SVM and random forest) have high predictive performance and are flexible enough to model multiple mechanisms of action. On the other hand, k-NN and SVM are not efficient for processing high-dimensional data without dimensionality reduction or prior selection of features (e.g., using genetic algorithms).

Using the test set in the best performing model (RF/RF combination), fluvoxamine was misclassified as fluoxetine (Figure 3). Fluvoxamine and fluoxetine belong in the same selective serotonin reuptake inhibitors family; therefore, the model may misclassify analogous compounds. For solving such misidentification, numerous transcripts from RNA sequencing are required. Furthermore, although the models mostly showed high accuracy, they were validated using a small test dataset. Thus, to recommend a particular model for aquatic toxicity monitoring, the models would require validation using a novel dataset. Additionally, further experiments are essential to obtain large datasets of similar families and broad exposure concentrations of toxicity compounds.

With increasing data dimensions, the amount of data required to provide a statistically significant result based on machine learning increases exponentially [24]. Recently, high quality and broad coverage RNA sequencing data of organisms under exposure to various contaminants has become available [12]. Therefore, RNA-seq data, as well as microarray data and exposure experiments, including abiotic and biotics factors, can impact the development of more comprehensive aquatic monitoring systems. In addition, deep learning approaches, such as artificial neural networks (ANN), using RNA-seq data, can improve performance [34]. Further research on these approaches, including a large data volume, will allow effective predicting of environmental factors that cause environmental problems in aquatic ecosystems, such as toxic inorganic compounds and nano/micro-plastics. In addition, we intend to establish models by using transcriptome profiles of various organisms that are mainly utilized for environmental monitoring in aquatic ecosystems. Finally, we aim to develop models designed to work over a wide range of experimental conditions by conducting larger studies that represent a much wider fraction and more complex spectrum of concentrations and time points of toxic substances.

## 5. Conclusions

In this study, we constructed an efficient model to identify toxic organic compounds in water, using a combination of machine learning-based feature-selection methods and nine classification algorithms. To evaluate linear, nonlinear, and ensemble classifiers, feature-selection methods, removing redundant features, and feature-ranking selection methods, based on three algorithms, were applied. We assessed the models with potential for building a model based on gene expression data. The model built in this study will be verified for its applicability in the field in the future. A portable sequencer will be used to analyze the transcriptome profile in the field, and the data will be trained on the model built through this study, which will be used for environmental monitoring in the field. This study is a preliminary investigation based on ML that can inform further research on NGS RNA sequencing data, and is potentially useful for aquatic environmental monitoring.

## Figures and Tables

**Table 1:** Summary of toxicity organic compounds in this study.

GEO Accession	Toxicity Organic Compounds
GSE43564	Atrazine, Acrylonitrile, Beta-benzene-hexachloride, Bifenthrin, Bis2-ethylhexyl-phthalate, Chlorpyrifos, Chloroform, Diazinon, Dichlorobenzene, Lamda-Cyhalothrin, Parathion, Phenol, Permethrin, Toluene, Trichloroethylene, 2-chloroethyl-vinyl-ether
GSE55132	Tris(2-butoxyethyl) phosphate (TBEP)
GSE43960	2,4,6-trinitrotoluene (TNT)
GSE45053	Acetone, Fluvoxamine, Fluoxetine, Nonylphenol (from Adult)

**Table 2:** The three algorithms for feature-ranking by importance used in this study.

Algorithm	Feature-Ranking Algorithm	Abbreviation
Artificial neural network	Learning Vector Quantization	LVQ
Ensemble	Random Forest	RF
Nonlinear	Support Vector Machines with a Linear kernel	SVML

**Table 3:** The nine classification algorithms used in this study.

Algorithm	Classification Algorithm	Abbreviation
Linear	Linear Discriminant Analysis	LDA
Nonlinear	Classification And Regression Trees K-nearest neighbors Support Vector Machines with a Linear kernel	CART Knn SVML
Ensemble	Random Forest Boosted C5.0 Gradient Boosting Machine eXtreme Gradient Boosting with tree eXtreme Gradient Boosting with DART booster	RF C5.0 GBM xgbTree xgbDART

## Data Availability

The data presented in this study are available within the article. If required, any additional data is available on request from the authors.

## Supplementary Materials

The following supporting information can be downloaded at: https://www.mdpi.com/article/10.3390/life12091443/s1, Table S1: Sublethal concentrations of 22 toxic organic compounds used in this study; Table S2: Complete dataset (total 13,481 features) used for machine learning; Table S3: Remaining 9423 predictors (features) after removal of redundant features; Table S4: Genes (features) ranked by importance using three algorithms; Table S5: Toxic organic compounds in confusion matrix using the RF/RF combination.
