# Supplementary material for: Machine Learning Models for Identification and Prediction of Toxic Organic Compounds Using Daphnia magna Transcriptomic Profiles
Source: Life (Basel). 2022 Sep 16;12(9):1443. doi: 10.3390/life12091443 (PMC9503646; doi:10.3390/life12091443)
Supplement: Supplementary file 1 [file life-12-01443-s001.zip › Supplementary materials.pdf]

Supplementary Table S1. Sublethal concentrations of 22 toxic organic compounds used in this study.

Organic compounds	Concentrations
2-chloroethyl-vinyl-ether	17.3 mg/L
Acetone	0.1 mL/L
Acrylonitrile	1.27 mg/L
Atrazine	2.07 mg/L
Beta-benzene-hexachloride	146 mg/L
Bifenthrin	0.0001 mg/L
Bis2-ethylhexyl-phthalate	0.039 mg/L
Chloroform	7.47 mg/L
Chlorpyrifos	0.00129 mg/L
Diazinon	0.0003 mg/L
Dichlorobenzene	1.51 mg/L
Fluvoxamine	0.01 mg/L
Fluoxetine	0.04 mg/L
Lamda-Cyhalothrin	0.0006 mg/L
Nonylphenol	0.02 mg/L
Parathion	0.00226 mg/L
Permethrin	0.00041 mg/L
Phenol	0.79 mg/L
Tris(2-butoxyethyl) phosphate (TBEP)	0.147 mg/L
2,4,6-trinitrotoluene (TNT)	1.85 mg/L
Toluene	3.58 mg/L
Trichloroethylene	2.49 mg/L

Supplementary Table S4. Genes (features) ranked by importance using three algorithms.

Learning Vector Quantization (LVQ)	Random Forest (RF)	Support Vector Machines with a Linear kernel (SVML)
DM03069	DM08825	DM03104
DM02384	DM08344	DM07830
DM00200	DM01585	DM12988
DM08718	DM09934	DM06333
DM09941	DM05612	DM10161
DM02148	DM03635	DM10057
DM01848	DM11516	DM00366
DM08097	DM04358	DM00978
DM14984	DM07257	DM05296
DM09156	DM07979	DM14215
DM06805	DM10434	DM11455
DM04984	DM11945	DM11502
DM01226	DM11139	DM15008
DM12308	DM04627	DM03273
DM10048	DM00637	DM00549
DM07956	DM15036	DM09188
DM11327	DM14324	DM09464
DM09472	DM02801	DM13286
DM12353	DM01012	DM06189
DM03700	DM05025	DM09347

Supplementary Table S5. Toxic organic compounds in confusion matrix using the RF/RF combination.

Number	Toxic organic compounds
1	2-chloroethyl-vinyl-ether
2	Acetone
3	Acrylonitrile
4	Atrazine
5	Beta-benzene-hexachloride
6	Bifenthrin
7	Bis2-ethylhexyl-phthalate
8	Chloroform
9	Chlorpyrifos
10	Diazinon
11	Dichlorobenzene
12	Fluvoxamine
13	Fluoxetine
14	Lamda-Cyhalothrin
15	Nonylphenol
16	Parathion
17	Permethrin
18	Phenol
19	Tris(2-butoxyethyl) phosphate
20	2,4,6-trinitrotoluene
21	Toluene
22	Trichloroethylene
